# Hyperactivation and enhanced cytotoxicity of reduced CD8^+^ gamma delta T cells in the intestine of patients with Crohn’s disease correlates with disease activity

**DOI:** 10.1186/s12865-024-00606-2

**Published:** 2024-02-09

**Authors:** Tao Zhu, Linlin Zhu, Caixia Sheng, Danju Wu, Qianru Gu, Zhinong Jiang, Jiaqi Xu, Guoxiang Fu, Yujie Jiang

**Affiliations:** 1 Department of Pathology, Sir Run Run Shaw Hospital Affiliated with Zhejiang University School of Medicine, Hangzhou, 310000 China; 2 Department of Gastroenterology, Sir Run Run Shaw Hospital Affiliated with Zhejiang University School of Medicine, Hangzhou, 310000 China

**Keywords:** CD8^+^ gamma delta T cell, Crohn’s disease, Immune characteristics, Disease activity

## Abstract

**Background and aims:**

We aimed to investigate the immune characteristics of intestinal CD8^+^ gamma delta T (CD8^+^ γδ T) cells in Crohn’s disease (CD) and their correlation with disease activity.

**Methods:**

The study cohorts included 21 CD patients and 21 healthy individuals. CD8^+^ γδ T cells were isolated from human ileal mucosa for detection by flow cytometry. The activation or inhibition status of cells was detected by detecting the expression of activation marker HLA-DR and the immunosuppressive molecule PD-1 on cells. The cytotoxicity of cells was assessed by detecting the expression of cytotoxic molecules (Perforin, Granzyme B, and TRAIL) in cells. Ratios of investigated cells were calculated as prediction factors by receiver operating characteristic curve (ROC) analysis.

**Results:**

The study revealed a reduction in intestinal CD8^+^ γδT cells among active CD patients, with a more pronounced reduction observed in moderately active patients compared to mildly active patients. Moreover, active CD patients exhibited heightened activation levels in their intestinal CD8^+^ γδT cells, whereas the activation was comparatively weakened in moderately active patients compared with mildly active patients. Additionally, the cytotoxicity of intestinal CD8^+^ γδT cells was enhanced solely in mildly active patients, while it was impaired in moderately active patients compared with mildly active patients. Furthermore, HLA-DR^+^ CD8^+^ γδT cell ratio, CD8^+^ γδT ratio, and CD8^+^ γδT count were identified as indicators in the diagnosis of active CD. Meanwhile, the ratios of Granzyme B^+^ CD8^+^ γδT cell and Perforin^+^ CD8^+^ γδT cell were identified as indicators that distinguish mildly moderately active CD cases.

**Conclusions:**

Intestinal CD8^+^ γδT was reduced in active CD patients, but their activation and cytotoxicity were enhanced. However, with increased disease activity, intestinal CD8^+^ γδ T cells became dysfunctional. CD-specific perturbations observed in various phenotypic markers in CD8^+^ γδ T cells can be used as indicators to assist in diagnosing CD patients.

**Supplementary Information:**

The online version contains supplementary material available at 10.1186/s12865-024-00606-2.

## Introduction

Crohn’s disease is a chronic inflammatory condition primarily affecting the gastrointestinal tract, potentially impacting any segment. This chronic disease may also manifest with extraintestinal symptoms, significantly diminishing the quality of life for patients and leading to substantial healthcare expenses [1, 2]. The incidence and prevalence of Crohn’s disease are higher in developed countries than in developing countries, and annual incidence rates are higher in urban areas than in rural areas. Notably, several countries in Asia have experienced rapid urbanization in recent years, coinciding with an upward trend in the annual incidence of Crohn’s disease [3, 4]. Crohn’s disease is believed to arise from the interplay between genetic susceptibility, environmental factors, and gut microbiota, leading to aberrant mucosal immune responses and impaired epithelial barrier function. Although current research has made significant advances in understanding the mechanisms underlying CD, the pathogenesis of the disease remains largely unknown [5, 6]. Recently, most studies on the pathogenesis of CD have focused on the dysregulation of innate and adaptive immune pathways [6–9]. Gammadelta (γδ) T cells are unconventional lymphocytes found primarily in skin and mucosal tissues and are regarded as the forefront of the immune defense against infections. They are vital in wound healing due to their immunomodulatory properties and the lack of major histocompatibility complex [MHC] restriction [10–13]. The antigen receptors of γδ T cells lack diversity and recognize antigens without MHC restrictions. They mainly recognize common antigenic components expressed by multiple pathogens presented by CD1 molecules, including glycolipids, glycoproteins of certain viruses, and phosphates of mycobacteria. Sugar and nucleotide derivatives, heat shock protein (HSP), etc. [14]. The γδ T cells can recognize a wide range of microorganisms, as well as infected or transformed host cells [15]. The cells contribute to direct cytotoxicity, involving both secretory and non-secretory pathways, i.e., the release of granzymes and perforins and the engagement of Fas and TNF-related apoptosis-inducing ligand [TRAIL], respectively [16–18]. The γδ T cells constitute a small proportion of circulating lymphocytes, primarily localized within the epithelium, and could account for up to 40% of colonic mucosal intraepithelial lymphocytes [IEL]. However, intestinal γδ IEL frequently comprises CD8^+^ [50% of γδ IEL] [19, 20]. Recent studies have unveiled that distinct subsets of γδ T cells within the human colon are subject to local regulation by the intestinal epithelium [21]. Recent studies have also demonstrated the importance of γδ T cells in the pathogenesis of CD [22]. However, the immune characteristics and clinical relevance of CD8^+^ γδT cells, the main component of intestinal γ δ T cells, in the intestine of CD patients are still unclear. Therefore, we undertook this study to investigate the immune characteristics of CD8^+^ γδ T cells in the intestinal tract of patients with CD, aiming to determine their clinical relevance. The study yielded significant and insightful findings.

## Materials and methods

### Characteristics of sample cohort

The advantage of this study is that since Crohn’s disease is still a rare disease in China, but this study obtained many precious tissue samples. Moreover, our screening criteria are very strict. We excluded surgical specimens for Crohn’s disease that were interfered by multiple factors and only collected biopsy specimens that met all screening criteria. The rigorous selection of specimens in this study also made the experimental results more convincing.

The fresh intestinal biopsy samples analyzed in this study included two groups of individuals: healthy controls (*n* = 21) and CD patients (*n* = 21). CD patients were diagnosed by endoscopy, histological criteria, radiological studies, and clinical parameters. All enrolled patients must be free of other autoimmune diseases, cancer, and infectious diseases such as tuberculosis or hepatitis B. Additionally, they should not have undergone intestinal surgery, used immunosuppressive drugs, received organ or bone marrow transplantation, or had blood transfusions within the past year. Only patients in the active phase were included, while those in remission were excluded. The disease activity of CD was assessed using the Crohn’s disease activity index (CDAI). A CDAI score of < 150 indicated disease remission, 150–220 indicated mild activity, 220–450 indicated moderate activity, and > 450 indicated severe activity. CD patients with intestinal biopsy samples were divided into subgroups of mildly active (*n* = 10) and moderately active (*n* = 11). In this study, samples of the enrolled cohorts were all obtained from the ileal mucosa. Intestinal biopsy samples from CD patients were obtained from ileal mucosa that was macroscopically inflamed but non-ulcerated. Intestinal biopsy samples of healthy controls were obtained from the ileal mucosa of subjects who underwent endoscopy but exhibited no endoscopic abnormalities. Intestinal biopsy samples required for the study were obtained from the Department of Gastroenterology, Sir Run Run Shaw Hospital, Zhejiang University School of Medicine, Hangzhou, China. This study was authorized by the Ethics Committee of Sir Run Run Shaw Hospital Affiliated with Zhejiang University School of Medicine (No. 2022 − 0293). The study was conducted according to the principles of the *Declaration of Helsinki*. All subjects signed written informed consent. Age and gender ratios did not differ significantly between the study cohorts. The clinical characteristics of the tissue donors are summarized in Table 1.

**Table 1:** Clinical characteristics of the tissue donors in the study

Group	HC	Mild CD	Moderate CD
**Case**	21	10	11
**Sex (male/female)**	12/9	8/2	6/5
**Age (years)**	43(16–73)	32(21–61)	25(15–44)
**CDAI score**	ND	189.7(161–214)	300.1(261.8-409.9)

Data are shown as median and range

ND: Not determined, HC: Healthy control, CD: Crohn’s Disease, CDAI: Crohn’s Disease Activity Index

### Sample acquisition and processing

Fresh intestinal mucosal tissues from CD patients and healthy individuals were obtained during endoscopy. The collected tissues were preserved in sterile PBS and promptly transported to the laboratory for further processing. Initially, the intestinal mucosal tissues were finely minced into small pieces. After incubation in Hank’s Balanced Salt Solution (HBSS) containing DTT and EDTA for 40 min, tissues were digested with Collagenase type IV and DNAse for 2 h on a shaker at 37 °C. The digested cell suspension was filtered through a 70-µm nylon mesh, and the filtrate was washed twice in sterile PBS. Finally, we isolated mononuclear cells from the ileal mucosa, including the intraepithelial compartment and lamina propria.

### Fluorescence-activated flow cytometry analysis

**Fig. 1:**
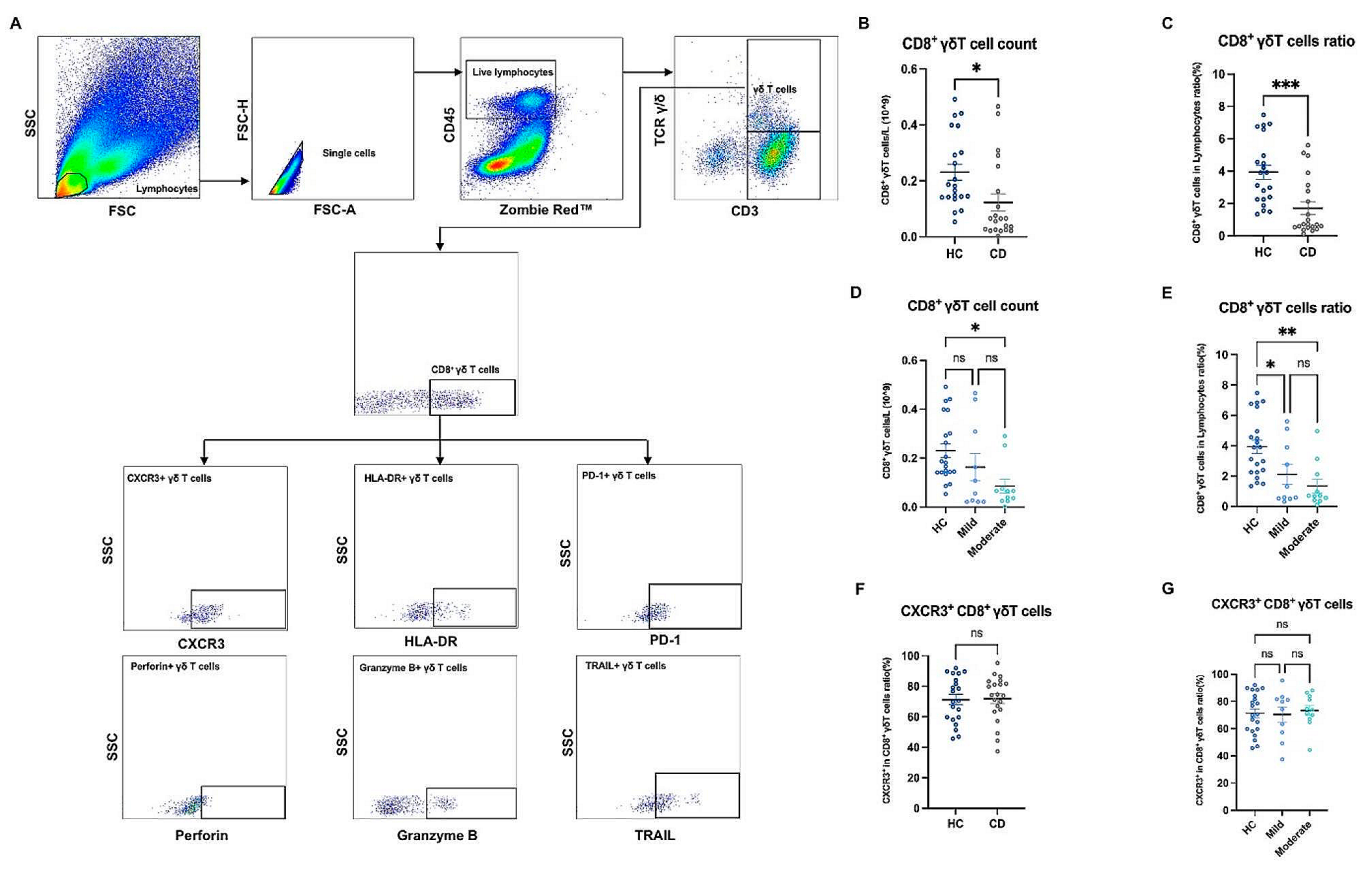
The decrease in intestinal CD8^+^ γδ T cells in active CD patients was influenced by the severity of disease activity. (**A**) A full gating of flow cytometry plots of the enrolled cohorts. (**B**) Pooled data compared the absolute count of CD8^+^ γδ T cells (CD3 + TCR γδ + CD8+) in the intestinal mucosa of HCs and active CD patients. (**C**) Pooled data compared the frequency of CD8^+^ γδ T cells in the intestinal mucosa of HCs and active CD patients. (**D**) Pooled data compared the absolute count of CD8^+^ γδ T cells in the intestinal mucosa from HCs, mildly active CD patients, and moderately active CD patients. (**E**) Pooled data compared the frequency of CD8^+^ γδ T cells in the intestinal mucosa from HCs, mildly active CD patients, and moderately active CD patients. (**F**) Pooled data compared the CXCR3 expression on CD8^+^ γδ T cells in the intestinal mucosa of HCs and active CD patients. (**G**) Pooled data compared the CXCR3 expression on CD8^+^ γδ T cells in the intestinal mucosa from HCs, mildly active CD patients, and moderately active CD patients. HCs: healthy controls, CD: Crohn’s disease, CXCR3: chemokine receptor marker. Data are mean ± SEM. *, *P* < 0.05; **, *P* < 0.01; ***, *P* < 0.001; ****, *p* < 0.0001

We utilized mononuclear cells derived from the ileal mucosa to assess the phenotypic and functional traits of CD8^+^ γδ T cells. The cells were subjected to cell surface staining, fixation, and permeabilization to enable intracellular staining. Cell staining was performed using the following fluorochrome-conjugated antibodies: Zombie Red™ Fixable Viability stain (BioLegend); Brilliant Violet 510™ anti-human CD45 mAb (Biolegend, USA), FITC anti-human CD3 mAb (Biolegend, USA), APC/Fire™ 750 anti-human CD8 mAb (Biolegend, USA), Brilliant Violet 421™ anti-human TCR γ/δ mAb (Biolegend, USA), Brilliant Violet 650™ anti-human CD183 (CXCR3) mAb (Biolegend, USA), Brilliant Violet 785™ anti-human HLA-DR mAb (Biolegend, USA), PE anti-human CD279(PD-1) mAb (Biolegend, USA), PE anti-human Perforin mAb (Biolegend, USA), Alexa Fluor® 700 anti-human/mouse Granzyme B Recombinant mAb (Biolegend, USA), PE/Cyanine7 anti-human CD253 (TRAIL) mAb (Biolegend, USA). Cell fluorescence intensity was determined by the DxFLEX flow cytometer (Beckman, USA). At least 100,000 events were collected for each sample. Flow cytometry results were analyzed by FlowJo software (Tree Star). The gating strategy is shown in Fig. 1A. To account for the continuous expression pattern of fluorescent markers on the mentioned partial antibodies (TCR γ/δ, CXCR3, HLA-DR, PD-1, Perforin, Granzyme B, and TRAIL), we employed Fluorescence Minus One (FMO) staining. The FMO staining results are included in the supplementary material (Fig. S1).

### Statistical analysis

Data on the graphs were expressed as the mean ± SEM for each group and were analyzed using GraphPad Prism software (v.9.4.0, Inc. San Diego, CA, USA). We used the Mann–Whitney U tests for single comparisons and two-way ANOVAs for multiple comparisons. The diagnostic value of each indicator was appraised using the receiver operation characteristic (ROC) curves. The 95% confidence interval was utilized to calculate the sensitivity, specificity and consistency, and the cut-off value was selected when the Jordan index was at its maximum. A two-sided *P* value < 0.05 was considered significant for all tests. The statistical significance was indicated as follows: ****p < 0.0001, ***p < 0.001, **p < 0.01, and *p < 0.05. Not significant: ns; *p* > 0.05.

## Results

### The decrease in intestinal CD8^+^ γδ T cells in active CD patients was influenced by the severity of disease activity

The absolute count of intestinal CD8^+^ γδ T cells in all enrolled subjects was analyzed by flow cytometry. The results revealed that the absolute count of intestinal CD8^+^ γδ T cells was significantly decreased in active CD patients compared to HCs (Fig. 1B). We then assessed the frequency of intestinal CD8^+^ γδ T cells in HCs and active CD patients. Our findings demonstrated a significant decrease in the frequency of intestinal CD8^+^ γδ T cells in active CD patients compared with HCs (Fig. 1C). These data strongly indicate a reduction of intestinal CD8^+^ γδ T cells in active CD patients.

To further understand the relationship between the decreased level of intestinal CD8^+^ γδ T cells in active CD patients and the varying degrees of disease activity in patients, we further investigated the absolute count and frequency of intestinal CD8^+^ γδ T cells in CD patients of varying degrees of disease activity. The results revealed that compared with HCs, the absolute count of intestinal CD8^+^ γδ T cells decreased only in moderately active CD patients and not in mildly active CD patients (Fig. 1D). Compared with HCs, the frequency of intestinal CD8^+^ γδ T cells in mildly and moderately active CD patients decreased. Furthermore, the decrease was more significant in moderately active CD patients (Fig. 1E). These data suggested that the reduction of intestinal CD8^+^ γδ T cells was more pronounced in moderately active CD patients as the degree of disease activity increased.

To understand the reason for the decrease in intestinal CD8^+^ γδ T cells of active CD patients, we analyzed the expression of the chemokine receptor CXCR3 on these cells. The purpose of this investigation was to assess the migratory potential of intestinal CD8 + γδ T cells. We found no significant difference in the expression of CXCR3 on intestinal CD8^+^ γδ T cells in active CD patients compared with HCs (Fig. 1F). In addition, there was no significant difference in the expression of CXCR3 on intestinal CD8^+^ γδ T cells among active CD patients with varying degrees of disease activity (Fig. 1G), suggesting that the migratory potential of intestinal CD8^+^ γδ T cells was unaltered in active CD patients regardless of disease activity.

### Immune activation of intestinal CD8^+^ γδT cells in active CD patients was affected by the varying degrees of disease activity

**Fig. 2:**
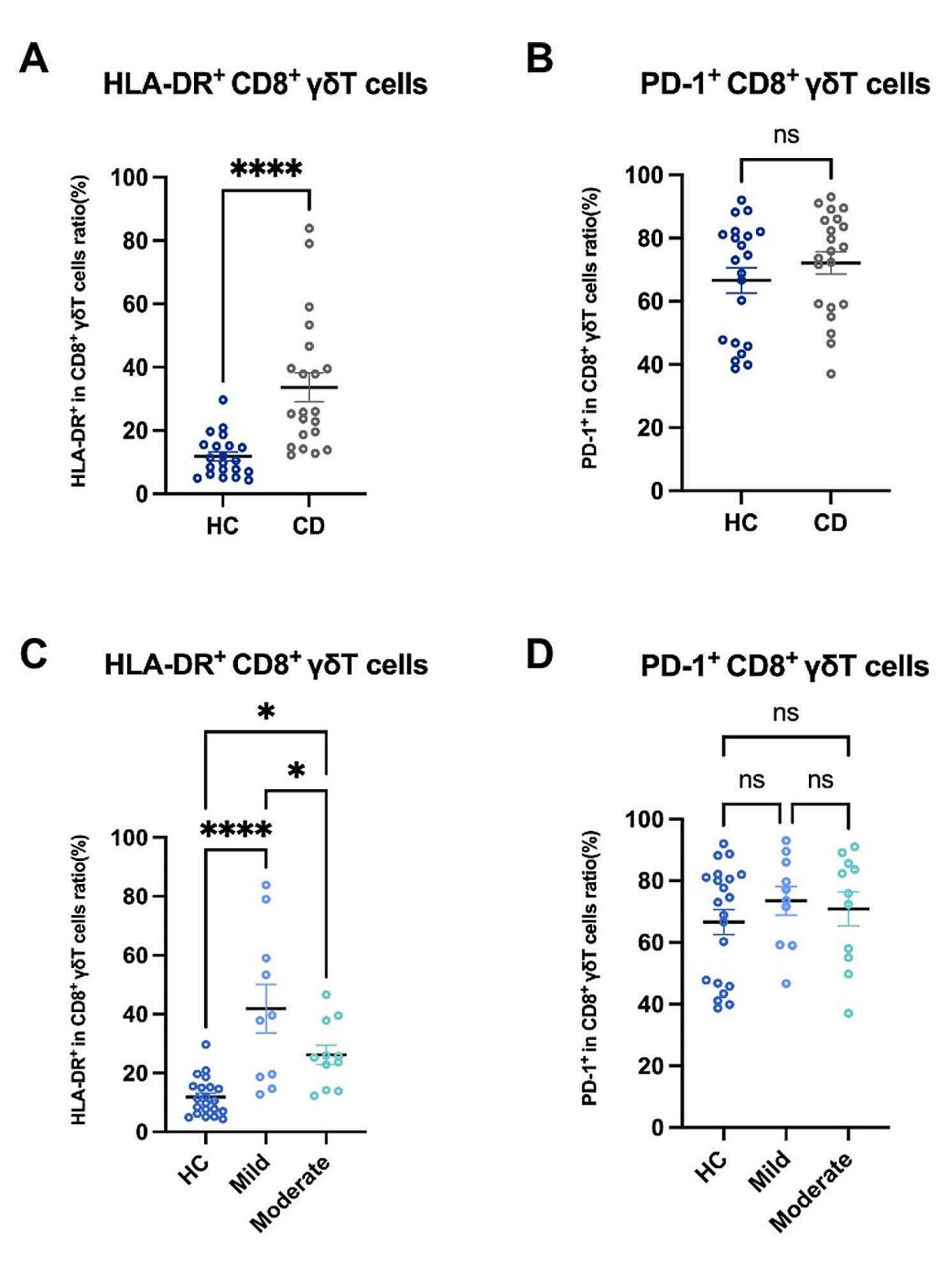
Immune activation of intestinal CD8^+^ γδT cells in active CD patients was affected by the varying degrees of disease activity. (**A**) Pooled data compared the HLA-DR expression on CD8^+^ γδ T cells in the intestinal mucosa of HCs and active CD patients. (**B**) Pooled data compared the PD-1 expression on CD8^+^ γδ T cells in the intestinal mucosa of HCs and active CD patients. (**C**) Pooled data compared the HLA-DR expression on CD8^+^ γδ T cells in the intestinal mucosa from HCs, mildly active CD patients, and moderately active CD patients. (**D**) Pooled data compared the PD-1 expression on CD8^+^ γδ T cells in the intestinal mucosa from HCs, mildly active and moderately active CD patients. HLA-DR: activation marker, PD-1: immunosuppressive molecule. Data are mean ± SEM. *, *P* < 0.05; **, *P* < 0.01; ***, *P* < 0.001; ****, *p* < 0.0001

We assessed whether the immune status of intestinal CD8^+^ γδ T cells in active CD patients was activated or suppressed by detecting the activation marker HLA-DR and the immunosuppressive molecule PD-1. Compared with HCs, we found that HLA-DR expression increased in intestinal CD8^+^ γδ T cells in active CD patients (Fig. 2A). Furthermore, there was no significant difference in the PD-1 expression in intestinal CD8^+^ γδ T cells in active CD patients compared with HCs (Fig. 2B). These findings suggest that intestinal CD8^+^ γδ T cells in active CD patients exhibited an immune activation status.

To gain a more comprehensive understanding of the association between the immune status of intestinal CD8^+^ γδ T cells in patients with active CD and the severity of disease activity, we conducted further investigations into the immune status of intestinal CD8^+^ γδ T cells in CD patients with varying degrees of disease activity. The results showed that HLA-DR expression was upregulated on intestinal CD8^+^ γδT in mildly and moderately active CD patients compared with HCs. However, compared with mildly active CD patients, the expression of HLA-DR decreased on intestinal CD8^+^ γδT in moderately active CD patients (Fig. 2C). In addition, we found no significant difference in the PD-1 expression on intestinal CD8^+^ γδ T cells in three groups (Fig. 2D). These data indicate that although intestinal CD8^+^ γδ T cells were highly activated in mildly and moderately active CD patients compared with HCs, their activation was more pronounced in mildly active CD patients. As disease activity increased, activation of intestinal CD8^+^ γδ T cells in moderately active CD patients weakened compared with that of mildly active CD patients.

### Enhanced cytotoxicity of intestinal CD8^+^ γδT cells in active CD patients was associated with disease activity degrees

**Fig. 3:**
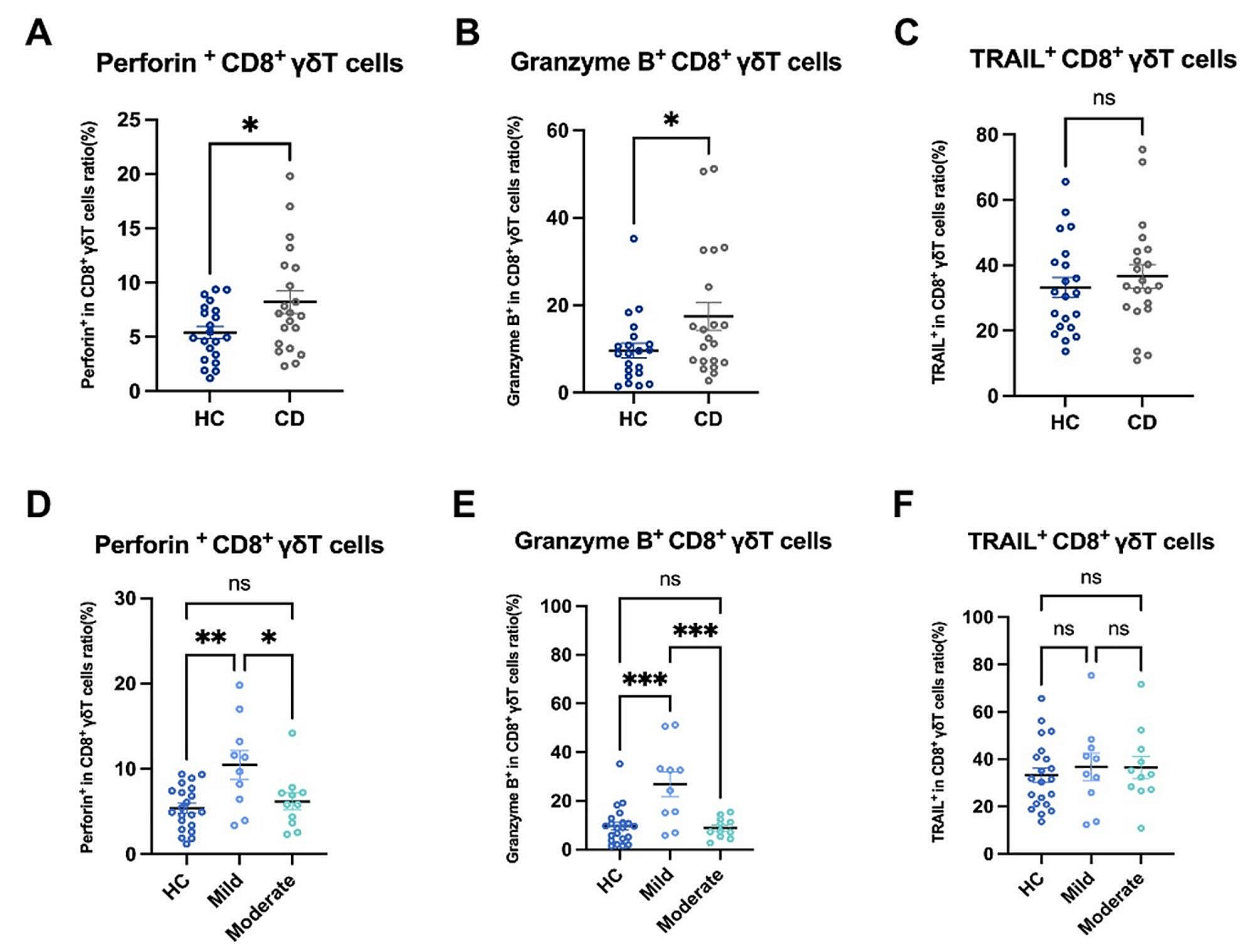
Enhanced cytotoxicity of intestinal CD8^+^ γδT cells in active CD patients was associated with disease activity degrees. (**A-C**) Pooled data compared the expression of cytotoxic molecules (Perforin, Granzyme B, and TRAIL) on CD8^+^ γδ T cells in the intestinal mucosa of HCs and active CD patients. (**D-F**) Pooled data compared the expression of cytotoxic molecules (Perforin, Granzyme B, and TRAIL) on CD8^+^ γδ T cells in the intestinal mucosa from HCs, mildly active CD patients, and moderately active CD patients. Perforin, Granzyme B, and TRAIL: cytotoxic molecules. Data are mean ± SEM. *, *P* < 0.05; **, *P* < 0.01; ***, *P* < 0.001; ****, *p* < 0.0001

To further understand the immune function of CD8^+^ γδT cells in active CD patients, we examined the expression of cytotoxic molecules (Perforin, Granzyme B, and TRAIL) in CD8^+^ γδT to examine the cytotoxicity of CD8^+^ γδT cells. The results showed that the expression of cytotoxic molecules (Perforin and Granzyme B) was significantly increased in intestinal CD8^+^ γδT cells in active CD patients compared with HCs (Fig. 3A, B). Furthermore, we found no significant difference in the TRAIL on intestinal CD8^+^ γδ T cells in active CD patients compared to HCs (Fig. 3C). These results suggest the enhanced cytotoxicity of intestinal CD8^+^ γδ T cells in active CD patients.

To understand the relationship between the immune function of intestinal CD8^+^ γδ T cells in active CD patients and the different degrees of disease activity in patients, we further investigated the immune function of intestinal CD8^+^ γδ T in CD patients of varying degrees of disease activity. We found that the expression of cytotoxic molecules (Perforin and Granzyme B) was significantly increased in intestinal CD8^+^ γδ T cells in mildly active CD patients compared with HCs. However, the expression did not differ significantly in moderately active CD patients. Compared with mildly active CD patients, the expressions of cytotoxic molecules (Perforin and Granzyme B) in intestinal CD8^+^ γδT cells of moderately active CD patients were significantly decreased (Fig. 3D, E). In addition, we found no significant difference in the expression of TRAIL on intestinal CD8^+^ γδ T cells in three groups (Fig. 3F). These data imply that intestinal CD8^+^ γδ T cells had enhanced cytotoxicity only in mildly active CD patients.

### ROC curve analysis of CD8^+^ γδ T cells in active CD patients

**Fig. 4:**
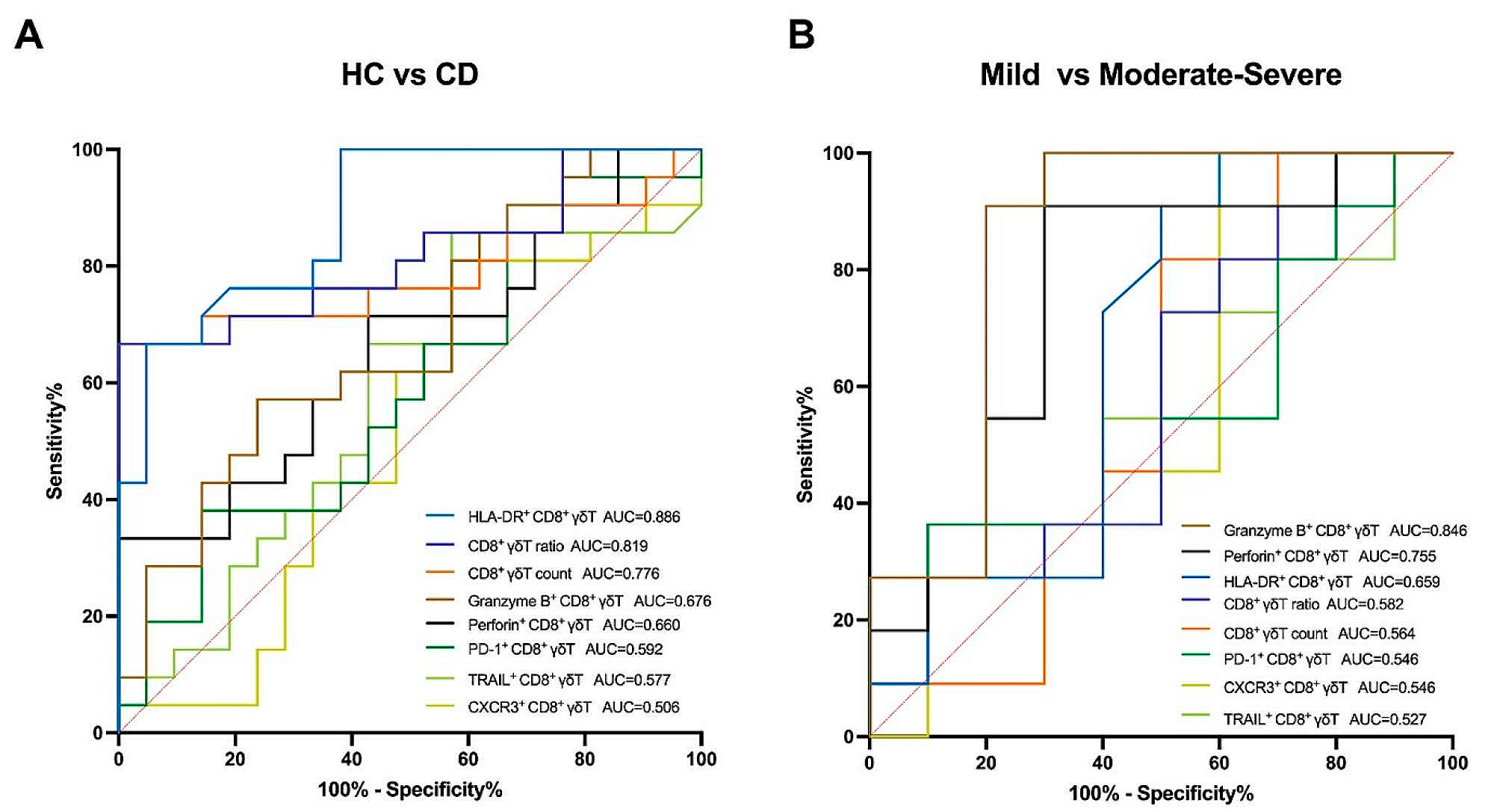
ROC curve analysis of CD8^+^ γδ T cells in active CD patients. (**A**) To distinguish between healthy controls and active CD cases. HLA-DR^+^ CD8^+^ γδT cell ratio (AUC, 0.886), CD8^+^ γδT ratio (AUC, 0.819), and CD8^+^ γδT count (AUC, 0.776) showed the best predictive value for active CD. (**B**) To distinguish between mildly active CD cases and moderately-severely active CD cases. Granzyme B^+^ CD8^+^ γδT cell ratio (AUC, 0.846) and Perforin^+^ CD8^+^ γδT cell ratio (AUC, 0.755) showed a good value to predict the moderately-severely active CD cases. AUC is an area under the curve

Based on ROC curve analysis, we found that HLA-DR^+^ CD8^+^ γδT cell ratio (AUC, 0.886), CD8^+^ γδT ratio (AUC, 0.819), and CD8^+^ γδT count (AUC, 0.776) exhibited remarkable predictive value for identifying active CD cases, with the high specificity of 95.24%, 100%, and 95.24% and sensitivity of 66.67%, 66.67%, and 66.67%, respectively (Fig. 4A). We utilized parameters that exhibited distinct expression levels in intestinal CD8^+^ γδT cells among patients with varying degrees of disease activity to predict the disease activity in patients with active CD. The results showed that Granzyme B^+^ CD8^+^ γδT cell ratio(AUC, 0.846) and Perforin^+^ CD8^+^ γδT cell ratio (AUC, 0.755) showed a good value to predict the moderately-severely active CD cases with the specificity of 80% and 70% and sensitivity of 90.91% and 90.91%, respectively (Fig. 4B). Meanwhile, other parameters were not statistically significant. (Detailed data are listed in Table 2).

**Table 2:** Receiver operation characteristic curve parameters for active CD diagnosis

Parameters	AUC	*P*	95% CI	Cut-off value	Jordan index	sensitivity	specificity
**HLA-DR** ^**+**^ **CD8** ^**+**^ **γδT**	0.886	< 0.0001	0.7886–0.9823	> 21.90	0.6191	66.67%	95.24%
**CD8** ^**+**^ **γδT ratio**	0.819	0.0004	0.6847–0.9525	< 1.325	0.6667	66.67%	100%
**CD8** ^**+**^ **γδT count**	0.776	0.0022	0.6234–0.9276	< 0.08073	0.619	66.67%	95.24%
**Granzyme B** ^**+**^ **CD8** ^**+**^ **γδT** ^**C**^	0.846	0.0075	0.6544- 1.000	< 14.75	0.7091	90.91%	80%
**Perforin** ^**+**^ **CD8** ^**+**^ **γδT** ^**C**^	0.755	0.0486	0.5325–0.9766	< 8.015	0.6091	90.91%	70%

HLA-DR^+^ CD8^+^ γδT, CD8^+^ γδT ratio, and CD8^+^ γδT count refer to the ability of HLA-DR^+^ CD8^+^ γδT cell ratio, CD8^+^ γδT ratio, and CD8^+^ γδT count to distinguish between healthy controls and active CD cases. Granzyme B^+^ CD8^+^ γδT^C^ and Perforin^+^ CD8^+^ γδT^C^ refer to the ability of Granzyme B^+^ CD8^+^ γδT cell ratio and Perforin^+^ CD8^+^ γδT cell ratio to distinguish between mildly active CD cases and moderately-severely active CD cases.AUC, area under the curve; 95% CI, 95% confidence interval

## Discussion

Previous studies have established a significant correlation between the dysregulation of the intestinal immune environment and the aberrant intestinal inflammatory response observed in patients with CD. Most prior studies have primarily emphasized the role of conventional T cells in the context of CD. Recently, studies have found that unconventional γδT cells also play a role in the pathogenesis and progression of CD [22]. Moreover, CD8^+^ γδT is the main population of intestinal γδT cells, and its immune characteristics in the intestinal tract of CD patients and its correlation with disease activity are still unclear. Therefore, we focused on the immune characteristics of CD8^+^ γδT cells in the intestinal tract of CD patients and their correlation with the degree of disease activity in this study.

We first assessed the absolute count and frequency of CD8^+^ γδ T cells in the gut of active CD patients. The results showed that the absolute count and frequency of CD8^+^ γδ T cells in the gut of active CD patients were reduced, with a more pronounced reduction as the disease activity degrees increased. Previous research has also shown that the frequency of intestinal CD8^+^ γδ T cells in active IBD patients was significantly lower compared with HCs, correlated negatively with the degree of disease activity, and increased to normal levels as a result of anti-TNF-α therapy [23]. The γδ T cells express the CXCR3 receptor on their surface, which binds to local cell-produced chemokines in inflammatory lesions, thereby recruiting CXCR3^+^ γδ T cells to inflammatory lesions [24]. Similarly, our research found that CD8^+^ γδ T cells express CXCR3 receptors on the surface. To comprehend the underlying cause of the decrease in intestinal CD8^+^ γδ T cells among patients with active CD, we investigated the expression of the chemokine receptor CXCR3 on CD8^+^ γδ T cells. This analysis aimed to assess the migratory potential of intestinal CD8^+^ γδ T cells. We found that the migratory potential of intestinal CD8^+^ γδ T cells was not altered in patients with active CD regardless of disease activity, so the decrease in CD8^+^ γδ T cells was not due to migratory behavior but could be due to other causes. For example, as the intestinal inflammatory environment worsened, cell death increased and cell proliferation decreased.

We then assessed whether the immune status of intestinal CD8^+^ γδ T cells in active CD patients was activated or suppressed by examining the activation marker HLA-DR and the immunosuppressive molecule PD-1. We found intestinal CD8^+^ γδ T cells were in a state of immune activation in active CD patients. Additionally, we found that although intestinal CD8^+^ γδ T cells were highly activated in mildly and moderately active CD patients compared with HCs, intestinal CD8^+^ γδ T cell activation was relatively attenuated in moderately active CD patients compared with mildly active CD patients. The reason may be that in patients with low disease activity, the intestinal immune environment is not yet imbalanced, CD8^+^ γδ T cells can still exert anti-inflammatory effects, and the cells remain active. As disease activity continues to increase, the patient’s intestinal inflammatory environment becomes more and more serious. Imbalance in the intestinal immune environment leads to weakened activation of CD8^+^ γδ T cells.

One study reported that aggravation of intestinal inflammation by depletion/deficiency of gammadelta T cells in different types of IBD animal models [25]. Another study reported CD8^+^ γδ T cells showed negative correlation with disease activity [23]. These data collectively suggest that CD8^+^ γδ T cells play an anti-inflammatory role in the human intestinal mucosa. Conventional CD8^+^ αβ T cells constitute the major cytotoxic T cell population in vivo, whereas γδ T cells have been proven cytotoxic [26]. Recent discoveries have demonstrated the cytotoxic nature of CD8^+^ γδ T cells, implying their potential anti-inflammatory role in eliminating infected cells, tumor cells, or cells experiencing stress due to various factors, including inflammation [23]. Furthermore, lymphocytes may upregulate their cytotoxic potential in situations requiring greater cytotoxicity in the gut, i.e., infection, tumor, or inflammation. Cellular cytotoxicity plays a role in inducing epithelial cell apoptosis and maintaining homeostasis. So how exactly do highly activated CD8^+^ γδ T cells in the intestine of active CD patients play an “anti-inflammatory” role? We further investigated the cytotoxicity of intestinal CD8^+^ γδ T cells in active CD patients by examining the expression of cytotoxic molecules (Perforin, Granzyme B, and TRAIL) in intestinal CD8^+^ γδ T cells. We found intestinal CD8^+^ γδ T cells in active CD patients had enhanced cytotoxicity compared with HCs. We further studied the relationship between the cytotoxicity of intestinal CD8^+^ γδ T cells in active CD patients and the different degrees of disease activity. We found that intestinal CD8^+^ γδ T cells had enhanced cytotoxicity only in mildly active CD patients. As disease activity increased, the cytotoxicity of intestinal CD8^+^ γδ T cells in moderately active CD patients decreased relatively compared with mildly active CD patients. These findings have allowed us to identify the disparities in immune characteristics of intestinal CD8^+^ γδ T cells between healthy individuals and those with CD. Furthermore, we have also delineated the variations in immune characteristics of intestinal CD8^+^ γδ T cells among CD patients with varying degrees of disease activity. These insights offer a valuable theoretical foundation for the precise diagnosis of active CD and the timely and effective prediction of disease activity levels.

At present, clinicians cannot accurately diagnose based on existing laboratory indicators. Although pathological diagnosis is the gold standard, diagnosis takes too long (about one week). We still need to find diagnostic methods that are accurate, convenient and time-consuming to gain critical treatment time for patients. It only takes about 6 h to obtain results using flow cytometry detection of biological markers in cells in this study. In addition, it does not require a separate invasion to obtain the specimen, but a small piece of biopsy tissue can be obtained during the necessary pathological biopsy, so it is feasible. Therefore, we further analyzed the ROC curve and found that HLA-DR^+^ CD8^+^ γδT cell ratio (AUC, 0.886; Sp, 95.24%; Se, 66.67%), CD8^+^ γδT ratio (AUC, 0.819; Sp, 100%; Se, 66.67%) and CD8^+^ γδT count (AUC, 0.776; Sp, 95.24%; Se, 66.67%) exhibited a good value for assisting diagnosis of active CD. In addition, we found that Granzyme B^+^ CD8^+^ γδT cell ratio (AUC, 0.846; Sp, 80%; Se, 90.91%) and Perforin^+^ CD8^+^ γδT cell ratio (AUC, 0.755; Sp, 70%; Se, 90.91%) are valuable indicators to help distinguish mildly-moderately active CD cases.

In conclusion, this study found that intestinal CD8^+^ γδT was reduced in active CD patients, but their activation and cytotoxicity were enhanced. Further evaluation revealed that the intestinal CD8^+^ γδT cells in mildly active CD patients decreased, and their activation and cytotoxicity were enhanced, which might demonstrate the positive anti-inflammatory effects of CD8^+^ γδT cells in mildly active CD patients. However, as the disease activity levels increased, we observed a more pronounced reduction in intestinal CD8^+^ γδ T cells among moderately active CD patients compared to those with mild disease activity. Meanwhile, their activation and cytotoxicity were relatively attenuated. This characterization reveals that the anti-inflammatory effects of CD8^+^ γδT cells might be impaired with increasing disease activity of CD patients. In addition, we found that HLA-DR^+^ CD8^+^ γδT cell ratio, CD8^+^ γδT ratio, and CD8^+^ γδT count had a good value for assisting diagnosis of active CD, whereas the ratios of Granzyme B^+^ CD8^+^ γδT cell and Perforin^+^ CD8^+^ γδT cell were valuable indicators to help distinguish mildly-moderately active CD cases. These findings may provide a new perspective and theoretical basis for CD patients’ clinical diagnosis and immunotherapy.

### Electronic supplementary material

Below is the link to the electronic supplementary material.

Supplementary Material 1

## Data Availability

The data underlying this article are available in the article and its online supplementary material.

## References

[1] Baumgart DC, Sandborn WJ (2012). Crohn’s disease. Lancet (London England).

[2] Rocchi A, Benchimol EI, Bernstein CN, Bitton A, Feagan B, Panaccione R, Glasgow KW, Fernandes A, Ghosh S (2012). Inflammatory bowel disease: a Canadian burden of illness review. Can J Gastroenterol = J canadien de gastroenterologie.

[3] Molodecky NA, Soon IS, Rabi DM, Ghali WA, Ferris M, Chernoff G, Benchimol EI, Panaccione R, Ghosh S, Barkema HW, Kaplan GG (2012). Increasing incidence and prevalence of the inflammatory bowel diseases with time, based on systematic review. Gastroenterology.

[4] Ng SC, Tang W, Ching JY, Wong M, Chow CM, Hui AJ, Wong TC, Leung VK, Tsang SW, Yu HH, Li MF, Ng KK, Kamm MA, Studd C, Bell S, Leong R, de Silva HJ, Kasturiratne A, Mufeena MNF, Ling KL, Colitis Epidemiologic Study (ACCESS) Study Group (2013). Incidence and phenotype of inflammatory bowel disease based on results from the Asia-pacific Crohn’s and colitis epidemiology study. Gastroenterology.

[5] Boyapati R, Satsangi J, Ho GT. (2015). Pathogenesis of Crohn’s disease. F1000prime reports, 7, 44. 10.12703/P7-44. 10.12703/P7-44 PMC4447044 26097717

[6] de Souza HS, Fiocchi C (2016). Immunopathogenesis of IBD: current state of the art. Nat Rev Gastroenterol Hepatol.

[7] Goldberg R, Prescott N, Lord GM, MacDonald TT, Powell N (2015). The unusual suspects–innate lymphoid cells as novel therapeutic targets in IBD. Nat Rev Gastroenterol Hepatol.

[8] Pariente B, Mocan I, Camus M, Dutertre CA, Ettersperger J, Cattan P, Gornet JM, Dulphy N, Charron D, Lémann M, Toubert A, Allez M (2011). Activation of the receptor NKG2D leads to production of Th17 cytokines in CD4 + T cells of patients with Crohn’s disease. Gastroenterology.

[9] Geremia A, Biancheri P, Allan P, Corazza GR, Di Sabatino A (2014). Innate and adaptive immunity in inflammatory bowel disease. Autoimmun rev.

[10] Grossi CE, Ciccone E, Migone N, Bottino C, Zarcone D, Mingari MC, Ferrini S, Tambussi G, Viale O, Casorati G (1989). Human T cells expressing the gamma/delta T-cell receptor (TcR-1): C gamma 1- and C gamma 2-encoded forms of the receptor correlate with distinctive morphology, cytoskeletal organization, and growth characteristics. Proc Natl Acad Sci USA.

[11] Toulon A, Breton L, Taylor KR, Tenenhaus M, Bhavsar D, Lanigan C, Rudolph R, Jameson J, Havran WL (2009). A role for human skin-resident T cells in wound healing. J Exp Med.

[12] Workalemahu G, Foerster M, Kroegel C, Braun RK (2003). Human gamma delta-T lymphocytes express and synthesize connective tissue growth factor: effect of IL-15 and TGF-beta 1 and comparison with alpha beta-T lymphocytes. J Immunol (Baltimore Md : 1950).

[13] Zheng J, Liu Y, Lau YL, Tu W (2013). γδ-T cells: an unpolished sword in human anti-infection immunity. Cell Mol Immunol.

[14] Lawand M, Déchanet-Merville J, Dieu-Nosjean MC (2017). Key features of Gamma-Delta T-Cell subsets in Human diseases and their immunotherapeutic implications. Front Immunol.

[15] Hayday AC (2000). [gamma][delta] cells: a right time and a right place for a conserved third way of protection. Annu Rev Immunol.

[16] Vantourout P, Hayday A (2013). Six-of-the-best: unique contributions of γδ T cells to immunology. Nat Rev Immunol.

[17] Qin G, Mao H, Zheng J, Sia SF, Liu Y, Chan PL, Lam KT, Peiris JS, Lau YL, Tu W (2009). Phosphoantigen-expanded human gammadelta T cells display potent cytotoxicity against monocyte-derived macrophages infected with human and avian influenza viruses. J Infect Dis.

[18] Dieli F, Troye-Blomberg M, Ivanyi J, Fournié JJ, Krensky AM, Bonneville M, Peyrat MA, Caccamo N, Sireci G, Salerno A (2001). Granulysin-dependent killing of intracellular and extracellular Mycobacterium tuberculosis by Vgamma9/Vdelta2 T lymphocytes. J Infect Dis.

[19] Andreu-Ballester JC, García-Ballesteros C, Benet-Campos C, Amigó V, Almela-Quilis A, Mayans J, Ballester F (2012). Values for αβ and γδ T-lymphocytes and CD4+, CD8+, and CD56 + subsets in healthy adult subjects: assessment by age and gender. Cytometry B.

[20] Deusch K, Lüling F, Reich K, Classen M, Wagner H, Pfeffer K (1991). A major fraction of human intraepithelial lymphocytes simultaneously expresses the gamma/delta T cell receptor, the CD8 accessory molecule and preferentially uses the V delta 1 gene segment. Eur J Immunol.

[21] Di Barros M, Roberts R, Dart NA, Vantourout RJ, Jandke P, Nussbaumer A, Deban O, Cipolat L, Hart S, Iannitto R, Laing ML, Spencer-Dene A, East B, Gibbons P, Irving D, Pereira PM, Steinhoff P, Hayday A (2016). Epithelia Use Butyrophilin-like molecules to shape organ-specific γδ T cell compartments. Cell.

[22] Lo Presti E, Di Mitri R, Mocciaro F, Di Stefano AB, Scibetta N, Unti E, Cicero G, Pecoraro G, Conte E, Dieli F, Meraviglia S (2019). Characterization of γδ T cells in intestinal mucosa from patients with Early-Onset or Long-Standing Inflammatory Bowel Disease and their correlation with clinical status. J Crohn’s Colitis.

[23] Kadivar M, Petersson J, Svensson L, Marsal J (2016). CD8αβ + γδ T cells: a novel T cell subset with a potential role in inflammatory bowel disease. J Immunol (Baltimore Md : 1950).

[24] Poggi A, Zancolli M, Catellani S, Borsellino G, Battistini L, Zocchi MR (2007). Migratory pathways of gammadelta T cells and response to CXCR3 and CXCR4 ligands: adhesion molecules involved and implications for multiple sclerosis pathogenesis. Ann N Y Acad Sci.

[25] Kühl AA, Pawlowski NN, Grollich K, Loddenkemper C, Zeitz M, Hoffmann JC (2007). Aggravation of intestinal inflammation by depletion/deficiency of gammadelta T cells in different types of IBD animal models. J Leukoc Biol.

[26] Siegers GM, Lamb LS (2014). Cytotoxic and regulatory properties of circulating Vδ1 + γδ T cells: a new player on the cell therapy field?. Mol Therapy: J Am Soc Gene Therapy.

