# Supplementary material for: Hyperactivation and enhanced cytotoxicity of reduced CD8+ gamma delta T cells in the intestine of patients with Crohn’s disease correlates with disease activity
Source: BMC Immunol. 2024 Feb 9;25:15. doi: 10.1186/s12865-024-00606-2 (PMC10858568; doi:10.1186/s12865-024-00606-2)
Supplement: Supplementary file 1 — Supplementary Material 1 [file 12865_2024_606_MOESM1_ESM.docx]

**Supplementary Material**


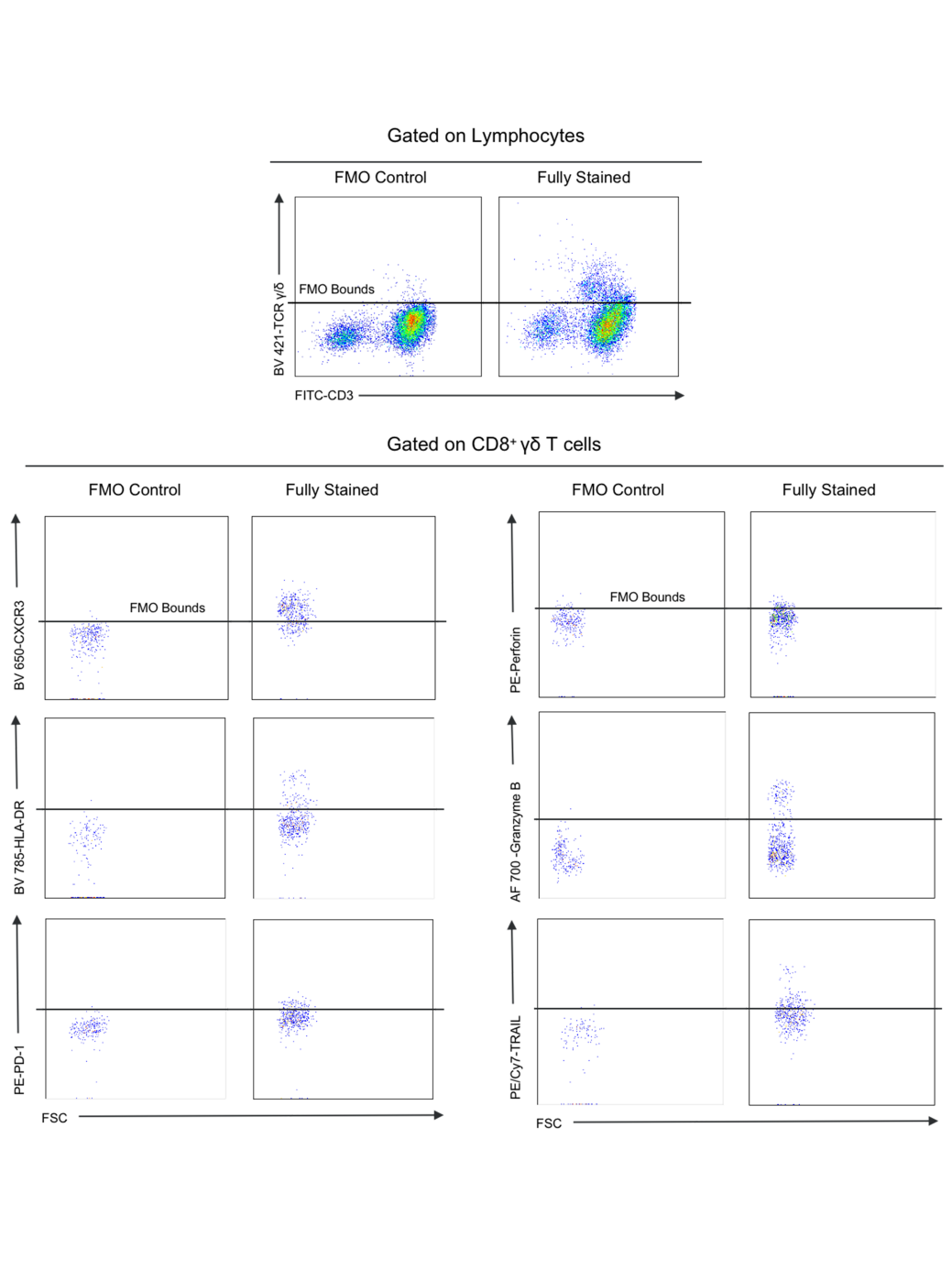


**Fig. S1. The Fluorescence Minus One staining results.** To account for the continuous expression pattern of fluorescent markers on the partial antibodies (TCR γ/δ, CXCR3, HLA-DR, PD-1, Perforin, Granzyme B, and TRAIL), we employed Fluorescence Minus One (FMO) staining.
